# Incidence of Chronic Spontaneous Urticaria Following Receipt of the COVID-19 Vaccine Booster in Switzerland

**DOI:** 10.1001/jamanetworkopen.2022.54298

**Published:** 2023-02-01

**Authors:** Olivier Duperrex, Francesco Tommasini, Yannick D. Muller

**Affiliations:** 1 Center for Primary Care and Public Health (Unisanté), Lausanne, Switzerland; 2 Lausanne University Hospital and University of Lausanne, Lausanne, Switzerland

## Abstract

This cohort study examines the association of the COVID-19 vaccine booster with chronic spontaneous urticaria in Swiss patients.

## Introduction

The messenger RNA (mRNA)–based vaccines Spikevax (Moderna) and Comirnaty (Pfizer-BioNTech) are the most widely distributed vaccines in Switzerland.^1^ Many chronic spontaneous urticaria (CSU) cases (recurrent wheals, angioedema, or both for more than 6 weeks^2^) have been observed after the booster dose.^3^ To assess whether a temporal association exists between COVID-19 vaccines and new-onset CSU, we compared the incidence rates of vaccine-related CSU in the canton of Vaud (CSU-Vaud) with all of Switzerland (CSU-Swiss).

## Methods

Sixteen local allergists helped identify eligible patients, who were then contacted through the Lausanne University Hospital. Patients were sent an online questionnaire link between April 14 and August 8, 2022. Because local allergists were eligible to identify but not include patients for this study, written informed consent was not required. The Commission Cantonale d’Éthique de la Recherche sur l’Être Humain approved the study. This study followed the STROBE reporting guideline.

We obtained the number of first booster doses given to the CSU-Vaud population (n = 298 813) and the CSU-Swiss population (n = 3 278 808) between December 1, 2021, and August 31, 2022, by brand. We calculated the crude incidence risk ratio of CSU per 100 000 persons having received a first booster dose and estimated the relative risk of CSU after the Moderna vs Pfizer-BioNTech booster (eMethods in Supplement 1).

## Results

Among 97 patients, 80 (56 [70%] female; median [IQR] age, 41 [35-49] years) agreed to participate and were assigned to the CSU-Vaud cohort. The CSU-Swiss cohort included 782 patients (446 [58%] female; median [IQR] age, 39 [33-48] years). In 72 patients (90%) in the CSU-Vaud cohort and 636 (81%) in the CSU-Swiss cohort, CSU started after the booster. The median (IQR) time between vaccination and CSU onset was 10 (8-12) days in the CSU-Vaud cohort and 11 (9-13) days in the CSU-Swiss cohort. Seventy-four cases (92%) in the CSU-Vaud and 77 (94%) in the CSU-Swiss cohort were associated with the Moderna vaccine (Table 1).

**Table 1.** Characteristics of the Study Participants^a^

Characteristic	All cases		After first booster	
	CSU-Vaud (n = 80)	CSU-Swiss (n = 782)	CSU-Vaud (n = 72)	CSU-Swiss (n = 636)
Sex				
Female	56 (70)	446 (58)	50 (69)	361 (58)
Male	24 (30)	319 (42)	22 (31)	263 (42)
Unknown	0	17	0	12
Age, median (IQR), y	41 (35-49)	39 (33-48)	41 (36-49)	39 (33-47)
Unknown	0	105	0	86
COVID-19 infection	25 (31)	NA	22 (31)	NA
Time between vaccine and COVID-19 infection, median (IQR), d	51 (18-89)	NA	48 (18-84)	NA
CSU symptoms worse after COVID infection	9 (11)	NA	8 (11)	NA
CSU active at data collection	65 (81)	650 (83)	62 (86)	564 (89)
Unknown	0	60 (7.6)	0	31 (4.8)
Duration of resolved CSU, median (IQR), d	86 (58-98)	98 (62-179)	82 (55-95)	NA
Vaccine that triggered CSU				
Moderna	74 (92)	77 (94)	69 (96)	607 (96)
Pfizer-BioNTech	6 (7.5)	47 (6.1)	3 (4.2)	25 (4.0)
Janssen	0	2 (0.3)	0	0
Unknown	0	6	0	4
Dose after which CSU appeared				
1 or 2	8 (10)	146 (19)	NA	NA
3	72 (90)	636 (81)	72 (100)	636 (100)
Time between vaccine and CSU, median (IQR), d^b^	10 (8-12)	11 (9-13)	10 (9-12)^a^	NA

Abbreviations: CSU, chronic spontaneous urticaria; CSU-Swiss, cohort of patients from Switzerland with CSU; CSU-Vaud, cohort of patients from canton of Vaud with CSU; NA, not applicable.

^a^ Data are presented as number (percentage) of patients unless otherwise indicated.

^b^ In 3 individuals, the time between vaccine and CSU could not be calculated.

In the CSU-Vaud cohort, 76 participants (95%) reported taking antihistamines (taken daily in 60). Of the 80 CSU-Vaud participants, 11 (14%) reported previous urticaria, 23 (29%) reported hay fever, and 9 (11%) reported drug allergies. At data collection, 25 patients (31%) reported a diagnosis of COVID-19 infection after vaccination, with a median (IQR) delay of 51 (18-89) days.

The overall crude incidence rate of CSU after a COVID-19 booster per 100 000 persons immunized with a booster was similar in the CSU-Vaud (n = 24) and CSU-Swiss (n = 19) cohorts (Table 2). Compared with the Pfizer-BioNTech vaccine, the relative risk of developing CSU after the Moderna vaccine was 20.8 (95% CI, 6.5-66.0) in the CSU-Vaud cohort and 16.1 (10.8-24.0) in the CSU-Swiss cohort (Table 2).

**Table 2.** Incidence of CSU After Receipt of the COVID-19 Booster

Vaccine	CSU-Vaud			CSU-Swiss		
	No. (%) of patients		Incidence rate per 100 000 (95% CI)^a^	No. (%) of patients		Incidence rate per 100 000 (95% CI)^a^
	Booster-Vaud	CSU-Vaud		Booster-Swiss	CSU-Swiss	
Pfizer-BioNTech	141 797 (48)	3 (4)	2.1 (0.6-6.7)	1 307 837 (40)	25 (4)	1.9 (1.3-2.9)
Moderna	157 016 (52)	69 (96)	43.9 (34.5-56.0)	1 970 971 (60)	607 (96)	30.8 (28.4-33.4)
Overall	298 813 (100)	72 (100)	24.1 (19.0-30.5)	3 278 808 (100)	632 (100)	19.3 (17.8-20.9)

Abbreviations: Booster-Swiss, cohort of patients from Switzerland who received the COVID-19 booster vaccine; Booster-Vaud, cohort of patients from canton of Vaud who received the COVID-19 booster vaccine; CSU, chronic spontaneous urticaria; CSU-Swiss, cohort of patients from Switzerland with CSU; CSU-Vaud, cohort of patients from canton of Vaud with CSU.

^a^ Crude incidence rate of CSU per 100 000 persons having received a first booster dose.

## Discussion

The results of this cohort study suggest an association between the booster dose of mainly the Moderna vaccine and new-onset CSU. However, this study has limitations. First, there is a selection bias for patients with CSU in relation to COVID-19 vaccines. Baseline data on the incidence of CSU, independent of COVID-19 vaccines, in the general population are not available in Switzerland. Second, a confounding association with the Omicron variant wave is also possible, although only 31% reported a confirmed infection in the CSU-Vaud cohort. Third, we could not adjust incidence rates because individual data on vaccination by brand, age, and sex were not available.

As a potential contributing mechanism warranting further investigations, our group previously showed that the Moderna vaccine had a greater association with positive skin and basophil activation tests results compared with the Pfizer-BioNTech vaccine.^4^ Alternatively, with the Moderna vaccines containing a higher dose of mRNA and being more immunogenic than the Pfizer-BioNTech vaccine,^5^ one could speculate that the booster nonspecifically triggered CSU in predisposed individuals.

These data should not discourage patients from being vaccinated. However, guidelines defining the eligibility and dosing for upcoming mRNA-based boosters are needed for patients with CSU after an mRNA-based COVID-19 vaccine.

## Notes

Supplement 1. **eMethods.** Supplemental Methods

Supplement 2.
Data Sharing Statement
