# Error in Table 1 and Results

**DOI:** 10.1001/jamanetworkopen.2023.9463

**Published:** 2023-04-05

In the Research Letter titled “Incidence of Chronic Spontaneous Urticaria Following Receipt of the COVID-19 Vaccine Booster in Switzerland,”^1^ published February 1, 2023, the number of participants in the CSU-Swiss cohort who received the Moderna vaccine was incorrectly written in the Results and Table 1 as 77 (94%); the correct number is 727 (93%). This article has been corrected.^1^
