# Supplementary material for: Incidence of Chronic Spontaneous Urticaria Following Receipt of the COVID-19 Vaccine Booster in Switzerland
Source: JAMA Netw Open. 2023 Feb 1;6(2):e2254298. doi: 10.1001/jamanetworkopen.2022.54298 (PMC9892951; doi:10.1001/jamanetworkopen.2022.54298)
Supplement: Supplement 1. — eMethods. Supplemental Methods [file jamanetwopen-e2254298-s001.pdf]

Supplementary Online Content

Duperrex O, Tommasini F, Muller YD. Incidence of chronic spontaneous urticaria following receipt of the COVID-19 vaccine booster in Switzerland. *JAMA Netw Open*. 2023;6(2):e2254298. doi:10.1001/jamanetworkopen.2022.54298

eMethods. Supplemental Methods

This supplementary material has been provided by the authors to give readers additional information about their work.

© 2023 Duperrex O et al. *JAMA Network Open.*

eMethods. Supplemental Methods

CSU-VAUD and CSU-SWISS cohorts

As there is no registry for CSU in the Canton de Vaud nor in Switzerland, we assembled the CSU-VAUD cohort and recorded the following variables from participants on an electronic data capture tools (REDCap) hosted at Unisanté (Lausanne, Switzerland): age, gender, timing of CSU onset, vaccines received, information relative to the UCT/UA7 scores, history of hay fever, drug allergies and history of COVID infection. We did not recorded information on omalizumab prescription.

Since the Canton de VAUD is only one of the 26 cantons forming Switzerland (estimated population size 0.8 million, 8.6 million in Switzerland, source https://www.bfs.admin.ch), we also obtained CSU incidence related to COVID-19 vaccines at the national level. I. Scholz (Pharmacovigilance Unit, Swissmedic) extracted CSU cases reported to Swissmedic from 2021-01-21 to 2022-08-31 (N=782). She analyzed and kindly shared the Swiss data respecting data protection rules.

Booster doses in VAUD and Switzerland

In Switzerland, the immunization protocol consisted of a primary series of two injections 3 to 4 weeks apart and a booster dose 4-6 months later. The Swiss regulatory agency for drugs and medical products (Swissmedic) approved the booster for the ≥18-year-old population by end of November 2021.

We obtained the number of first booster doses given from the public website of Federal Office for Public health.

Statistics

To acquire, recode and analyse the data, we used R v4.2.2, RStudio v2022.07.2+576, REDCapR v1.1.0, data.table v1.14.6, gtsummary v1.6.2, epiR v2.0.53.

We summarized categorical and dichotomous variable with absolute numbers and simple proportions. As the continuous variables presented here (age, days between vaccine and COVID, days between vaccine and CSU) had a non-normal distribution, we used median and interquatile ranges (IQR). We estimated the crude incidence risk ratio with the function *epiR::epi.2by()* which uses 'the exact method described by Kirkwood and Sterne (2003) and Juul (2004)', (https://rdrr.io/cran/epiR/man/epi.2by2.html). Adjustments of the incidence rate ratio for age and sex distribution were not possible as detailed data at national level by brand, gender and age was not publicly available.

© 2023 Duperrex O et al. *JAMA Network Open.*
