# Supplementary material for: Incidence of Chronic Spontaneous Urticaria Following Receipt of the COVID-19 Vaccine Booster in Switzerland
Source: JAMA Netw Open. 2023 Feb 1;6(2):e2254298. doi: 10.1001/jamanetworkopen.2022.54298 (PMC9892951; doi:10.1001/jamanetworkopen.2022.54298)
Supplement: Supplement 2. — Data Sharing Statement [file jamanetwopen-e2254298-s002.pdf]

Data Sharing Statement

Duperrex. Incidence of Chronic Spontaneous Urticaria Following Receipt of the COVID-19 Vaccine Booster in Switzerland. *JAMA Netw Open*. Published February 01, 2023. doi:10.1001/jamanetworkopen.2022.54298

Data
Data available: No
